# Increased Cortical-Limbic Anatomical Network Connectivity in Major Depression Revealed by Diffusion Tensor Imaging

**DOI:** 10.1371/journal.pone.0045972

**Published:** 2012-09-26

**Authors:** Peng Fang, Ling-Li Zeng, Hui Shen, Lubin Wang, Baojuan Li, Li Liu, Dewen Hu

**Affiliations:** 1 College of Mechatronics and Automation, National University of Defense Technology, Changsha, Hunan, People's Republic China; 2 Department of Psychiatry, First Affiliated Hospital, China Medical University, Shenyang, Liaoning, People's Republic China; Institution of Automation, CAS, China

## Abstract

Magnetic resonance imaging studies have reported significant functional and structural differences between depressed patients and controls. Little attention has been given, however, to the abnormalities in anatomical connectivity in depressed patients. In the present study, we aim to investigate the alterations in connectivity of whole-brain anatomical networks in those suffering from major depression by using machine learning approaches. Brain anatomical networks were extracted from diffusion magnetic resonance images obtained from both 22 first-episode, treatment-naive adults with major depressive disorder and 26 matched healthy controls. Using machine learning approaches, we differentiated depressed patients from healthy controls based on their whole-brain anatomical connectivity patterns and identified the most discriminating features that represent between-group differences. Classification results showed that 91.7% (patients = 86.4%, controls = 96.2%; permutation test, *p*<0.0001) of subjects were correctly classified via leave-one-out cross-validation. Moreover, the strengths of all the most discriminating connections were increased in depressed patients relative to the controls, and these connections were primarily located within the cortical-limbic network, especially the frontal-limbic network. These results not only provide initial steps toward the development of neurobiological diagnostic markers for major depressive disorder, but also suggest that abnormal cortical-limbic anatomical networks may contribute to the anatomical basis of emotional dysregulation and cognitive impairments associated with this disease.

## Introduction

Major depressive disorder (MDD), which has been linked to a 15% suicide rate among those suffering from the disorder, serious social problems and tremendous economic loss, both directly and indirectly, has been ranked by the World Health Organization as the number one reason why people file for disability benefits [1]. Although tremendous efforts have been made to understand the neuropsychology and etiology of depression, little is known about its pathogenesis. These days, magnetic resonance imaging (MRI) provides a powerful tool for exploring the neuropathology of this complex mental disorder [2]. For example, functional MRI (fMRI) studies have reported abnormalities in several specific brain areas in patients suffering from depression, including the amygdala [3], hippocampus [4], caudate, ventral striatum [5], orbitofrontal cortex (OFC) [6], prefrontal cortex [7], subgenual cingulate and thalamus [8]. In MDD patients, structural MRI studies using voxel-based morphometry (VBM) have shown alterations in gray matter volume of the hippocampus [9], anterior cingulate (ACC), OFC [10], right amygdala [11] and caudate [12].

The widely distributed functional and structural abnormalities found in the brains of MDD patients suggest that depression may be considered a multi-dimensional and systems-level mental disorder, which affects discrete but functionally integrated circuits, rather than dysfunction in one or more discrete brain regions [13]. Furthermore, the remaining normal brain that fails to maintain homeostatic emotional control in times of increased cognitive or somatic stress is believed to be associated with MDD [14]. Investigators even speculate that depression is caused by the dysfunction of coping mechanisms rather than lesioned brain areas [15]. Further evidence from functional connectivity studies of depressed patients reveal altered network connectivity in the limbic-cortical-striatal-pallidal-thalamic circuit (LCSPT) [1], the prefrontal-limbic network [16], the default-mode network (DMN) [8], [17], the cerebellar network [18], the cognitive control network and the affective network [19]; therefore, some researchers speculate that dysfunction in these circuits or networks can produce pathological emotional symptoms [1]. The anatomical basis of these disorder-related connectivity abnormalities both within and across different functional networks remains unclear.

In studies searching for anatomical changes in MDD, diffusion tensor imaging (DTI) has been suggested as a non-invasive method for detecting subtle changes in tissue microstructural organization [20]. There are two main research methods in DTI studies: the fractional anisotropy (FA) study and white matter (WM) tractography. As a measure of the directionality of diffusion anisotropy, FA has been widely used to investigate the WM abnormalities present in many mental disorders [2], [21], [22], [23]. It is nearly impossible, however, to interpret the precise physiological meaning of these observed changes because the changes in FA may result from alterations in axonal morphologic structure, in the interaxonal spacing of fiber bundles, and so on [20]. In contrast, WM tractography can be used to study connectivity between neural regions of interest (ROIs) and give rise to astonishing visualizations of brain circuitry. By using anatomical connectivity to investigate neural differences, several DTI studies have identified connectivity abnormalities in those who suffer from bipolar disorder [24], aging [25], etc. Nevertheless, anatomical connectivity is seldom utilized to investigate abnormal brain networks in patients with MDD.

In recent years, machine learning approaches have been increasingly used for brain image analysis [17], [26], [27], [28], [29] because they are capable of extracting stable patterns from neuroimaging data, finding significant neuroimaging-based biomarkers and identifying depressed patients from control participants at individual subject levels [26], [30]. Using adaptive regional elements and a linear support vector machine (SVM) classifier, Fan has even differentiated individuals with mild cognitive impairment from controls with a 100% classification rate [26]. Unfortunately, it is unclear whether machine learning approaches can extract whole-brain anatomical connectivity patterns to differentiate depressed patients from controls with a high level of accuracy.

Here, we hypothesized that there were significant anatomical-connectivity abnormalities in MDD. Furthermore, we speculated that the changed anatomical connectivity patterns could be used to differentiate depressed patients from controls on a case-by-case basis and may be considered a potential biomarker for MDD. To test these hypotheses, we first adopted DTI-based probabilistic tractography to reconstruct the tracts and to extract anatomical networks. Second, we used machine learning approaches to select the most discriminating connections. Finally, the selected connections were further discussed in terms of potential use as biomarkers for MDD.

## Results

### Whole brain inter-regional tractography

**Figure 1:**
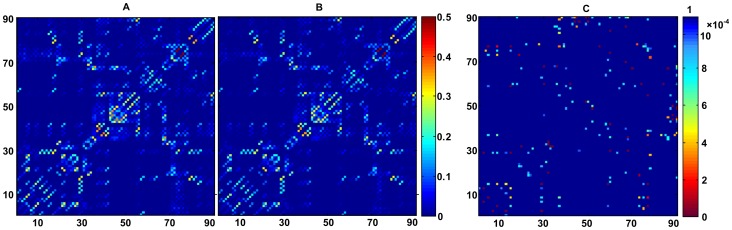
Mean connectivity strength matrix and significance matrix across populations. A: mean connectivity strength matrix of depressed patients. B: mean connectivity strength matrix of healthy controls. C: significance matrix, representing *t*-statistics for the significance of any differences across populations for all connections. Left color bar represents connectivity strength, while right color bar represents *p*-value. Results are indexed in 90×90 matrices. Symmetry is enforced.

The averaged connectivity matrix for each group was shown in Figure 1A and B. The mean strength for non-zero connectivities of the depressed patients was significantly higher (Mean+SD = 0.0499±0.0053) than that of controls (Mean+SD = 0.0412±0.0045), with *p* = 0.021. The significance level of the differences in the connectivity matrix between the two groups was presented in Figure 1C.

### Classification results and the most discriminating features

Fifty of the most discriminating features were selected for each fold in a leave-one-out cross-validation (LOOCV), with two-sample *t*-tests (TSTT), and the *p*-values of these features were all found to be lower than 0.001. Using the SVM classifier with a local linear embedding (LLE) algorithm, we obtained a classification rate of 91.7% (sensitivity 86.4%, specificity 96.2%; permutation test, *p*<0.0001) via LOOCV. Here, twenty-three local neighborhood points were chosen, and the number of intrinsic dimensions was reduced to fifteen in the LLE. Moreover, we trained the SVM classifier with a Gaussian radial basis kernel function, defined as 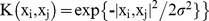, where x_i_ represented the *i*-th feature vector and 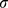 was set to be equal to 3.

**Figure 2:**
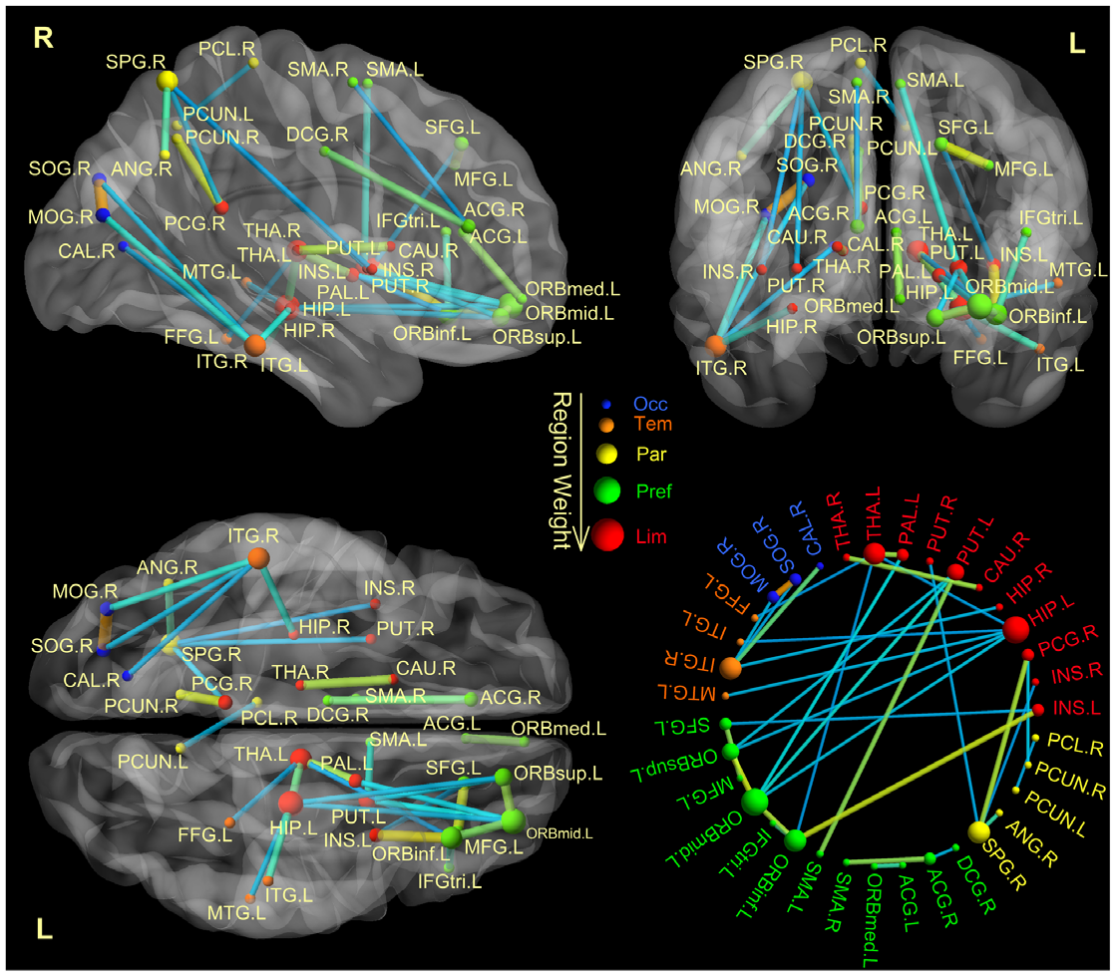
Region weights and distribution of the consensus anatomical connections. The consensus anatomical connections are displayed both on a surface rendering of the brain and in a circle. The thickness of connections adjusts according to their connectivity strength. The connectivity for either low or high values is color-coded in blue and orange. The diameter of a sphere represents the corresponding region weight of a ROI. The ROIs are color-coded according to brain areas (red, limbic cortex; green, prefrontal cortex; yellow, parental cortex; orange, temporal cortex; blue, occipital cortex). R = right hemisphere, L = left hemisphere. SFG = Superior Frontal; ORBsup = Superior Orbital Frontal; MFG = Middle Frontal; ORBmid = Middle Orbital Frontal; IFGtriang = Inferior Triangular Frontal; ORBinf = Inferior Orbital Frontal; SMA = Supplementary Motor Area; ORBsupmed = Medial Orbital Frontal; INS = Insula; ACG = Anterior Cingulate; DCG = Middle Cingulate; PCG = Posterior Cingulate; HIP = Hippocampus; CAL = Calcarine; SOG = Superior Occipital; MOG = Superior Occipital; FFG = Fusiform; SPG = Superior Parietal; ANG = Angular; PCUN = Precuneus; PCL = Paracentral Lobule; CAU = Caudate; PUT = Putamen; PAL = Pallidum; THA = Thalamus; MTG = Middle Temporal; ITG = Inferior Temporal. Brain networks are visualized using the BrainNet Viewer (http://www.nitrc.org/projects/bnv/).

Because the training data sets differed slightly from fold to fold in the LOOCV, the selected feature sets may also differ from fold to fold. Thirty-three features were included in each fold in the LOOCV, which may be viewed as the most discriminating features, named “consensus features” [29]. All of these consensus features exhibited increased connectivity in depressed patients, and they were primarily distributed in the cortical-limbic network. Furthermore, the cortical-limbic network, in which the consensus connections were distributed, could be sub-divided into the frontal-limbic, parietal-limbic and temporal-limbic networks. In addition, four connections were located in the temporal-occipital network, including connections from the right inferior temporal gyrus to the superior occipital, the middle occipital and the calcarine gyri (see Table 1). The region weight, which represents the relative contribution to identification, was denoted by its occurrence number in the consensus anatomical connections. In this study, the left OFC exhibited the greatest brain region weight out of all the consensus connections. The region weight and distribution of the consensus connections are shown in Figure 2.

**Table 1:** The consensus features (Thirty-three anatomical connections between two ROIs).

ROI A	ROI B	Network	Subnetwork	*p*-value
Superior Frontal Gyrus L	Middle Frontal Gyrus L	Cortical-Limbic	frontal-limbic	9.11E-05
Superior Frontal Gyrus L	Insula L	Cortical-Limbic	frontal-limbic	2.05E-04
Superior Orbital Frontal Gyrus L	Middle Orbital Frontal Gyrus L	Cortical-Limbic	frontal-limbic	6.49E-05
Superior Orbital Frontal Gyrus L	Hippocampus L	Cortical-Limbic	frontal-limbic	8.57E-05
Superior Orbital Frontal Gyrus L	Putamen L	Cortical-Limbic	frontal-limbic	3.39E-04
Middle Orbital Frontal Gyrus L	Inferior Orbital Frontal Gyrus L	Cortical-Limbic	frontal-limbic	2.16E-04
Middle Orbital Frontal Gyrus L	Hippocampus L	Cortical-Limbic	frontal-limbic	1.07E-04
Middle Orbital Frontal Gyrus L	Putamen L	Cortical-Limbic	frontal-limbic	2.02E-04
Middle Orbital Frontal Gyrus L	Pallidum L	Cortical-Limbic	frontal-limbic	3.57E-04
Inferior Triangular Frontal Gyrus L	Inferior Orbital Frontal Gyrus L	Cortical-Limbic	frontal-limbic	2.28E-04
Inferior Orbital Frontal Gyrus L	Insula L	Cortical-Limbic	frontal-limbic	6.85E-07
Inferior Orbital Frontal Gyrus L	Thalamus L	Cortical-Limbic	frontal-limbic	2.47E-04
Medial Orbital Frontal Gyrus L	Anterior Cingulate Gyrus R	Cortical-Limbic	frontal-limbic	3.52E-04
Anterior Cingulate Gyrus R	Middle Cingulate Gyrus R	Cortical-Limbic	frontal-limbic	3.92E-05
Supplementary Motor Area L	Putamen L	Cortical-Limbic	frontal-limbic	1.51E-04
Supplementary Motor Area R	Anterior Cingulate Gyrus R	Cortical-Limbic	frontal-limbic	1.44E-04
Hippocampus L	Thalamus L	Cortical-Limbic	frontal-limbic	1.83E-04
Caudate Nucleus R	Thalamus R	Cortical-Limbic	frontal-limbic	1.76E-04
Pallidum L	Thalamus L	Cortical-Limbic	frontal-limbic	3.46E-04
Insula R	Superior Parietal Gyrus R	Cortical-Limbic	parietal-limbic	2.26E-05
Posterior Cingulate Gyrus R	Superior Parietal Gyrus R	Cortical-Limbic	parietal-limbic	3.07E-04
Posterior Cingulate Gyrus R	Precuneus R	Cortical-Limbic	parietal-limbic	7.61E-05
Superior Parietal Gyrus R	Putamen R	Cortical-Limbic	parietal-limbic	1.15E-05
Superior Parietal Gyrus R	Angular R	Cortical-Limbic	parietal-limbic	3.97E-04
Precuneus L	Paracentral Lobule R	Cortical-Limbic	parietal-limbic	2.12E-05
Fusiform L	Thalamus L	Cortical-Limbic	temporal-limbic	4.73E-05
Hippocampus L	Middle Temporal Gyrus L	Cortical-Limbic	temporal-limbic	2.15E-04
Hippocampus L	Inferior Temporal Gyrus L	Cortical-Limbic	temporal-limbic	1.88E-04
Hippocampus R	Inferior Temporal Gyrus R	Cortical-Limbic	temporal-limbic	1.96E-04
Calcarine R	Inferior Temporal Gyrus R	Occipital-Temporal		2.20E-04
Superior Occipital Gyrus R	Middle Occipital Gyrus R	Occipital-Temporal		3.26E-04
Superior Occipital Gyrus R	Inferior Temporal Gyrus R	Occipital-Temporal		5.85E-05
Middle Occipital Gyrus R	Inferior Temporal Gyrus R	Occipital-Temporal		4.16E-04

Network shows the network to which the connection belongs. Subnetwork shows the subnetwork to which the connection belongs. A *p*-value indicates the mean *p*-value of the connection in a two-sample *t*-test. L represents the left hemisphere, while R represents the right hemisphere.

## Discussion

In this work, we adopted anatomical connectivity and machine learning approaches to study the whole-brain anatomical network differences in macroscopic neural tracts between depressed patients and controls. The classifier successfully distinguished the depressed patients from controls with an accuracy of 91.7% (permutation test, *p*<0.0001) and identified thirty-three of the most discriminating consensus connections. The altered anatomical connections were all increased in depressed patients and primarily distributed in the cortical-limbic network. In addition, four connections were located in the temporal-occipital network.

### Altered anatomical networks

#### Frontal-limbic network

The consensus connections were mainly distributed in the cortical-limbic network, which may be sub-divided into the frontal-limbic, parietal-limbic and temporal-limbic networks. The abnormal connectivity in the frontal-limbic network was in keeping with limbic-cortical-striatal-pallidal-thalamic circuits involved in mood regulation and cognition [1], [31], such as the connections between the OFC, basal ganglia, thalamus, hippocampus and insula. The OFC, which played a dominant role in the frontal-limbic network in this study, was mostly implicated in emotional processing, emotional regulation [32] and emotional response to stressors [33]. Furthermore, the OFC projects to the striatum, which then projects to the mediodorsal thalamic nucleus and then back to the OFC. Dysfunction in this circuit is hypothesized to bias information-processing in MDD in such a way that depressed individuals selectively attend to and remember affectively negative material [34]. In addition, several abnormal connections in the frontal-limbic network were located in the ventral system, including the insula, ventral striatum, ventral anterior cingulate gyrus and prefrontal cortex. The ventral system is involved in the identification of the emotional significance of a stimulus, the production of affective states and the automatic regulation of emotional responses [35]. Increased connectivity within the ventral system may result in a restricted emotional range, biased toward the perception of negative rather than positive emotions [35]. Because the structures in the frontal-limbic network are involved in emotional regulation, changes in any portion of the network could potentially result in depressed mood in MDD patients [31], [36], [37].

#### Parietal-limbic network

Altered connectivity in the parietal-limbic network was found to be related to the DMN in regions known to be involved in attention, cognition and self-referential activity [19], [38], including the superior parietal lobe, the insula, the posterior cingulate, and the precuneus. The superior parietal lobe is essentially related to the elaboration of somatosensory information [39] and selective attention [40]. In addition, the parietal and cingulate areas are involved in attentional, motivational, and emotional modulations of the sensorimotor functions [41]. Abnormal connectivity between the parietal and cingulate areas may lead to biased attention and restricted emotions in MDD. Furthermore, increased connectivity in the frontal-limbic and parietal-limbic networks was consistent with the limbic-cortical model proposed by Mayberg [13], [14], [42], [43]. The limbic-cortical model is found to be critical in the integrated regulation of mood, associated motor, cognitive and somatic behaviors [13], [44]. Altered connectivity in these two networks may lead to a restricted emotional range with a bias towards the perception of negative emotions [35].

#### Temporal-limbic network

Episodic memory seems to be the main feature of cognitive functioning that is vulnerable to the negative effects of MDD, while temporal lobe and hippocampus lesions in the temporal-limbic network typically disrupt episodic memory and cognition [45]. DTI studies reported a correlation between memory and learning impairment and abnormality in the hippocampal and temporal cortex [46]. Increased connectivity between hippocampal and the temporal gyrus may disrupt memory and cognition in MDD. These findings implied that the anatomical abnormalities in the temporal-limbic network may contribute to some types of cognitive impairment seen in major depression [47]. Taken together, it was tempting to speculate that the imbalanced anatomical connectivity in the cortical-limbic network could result in mood and cognitive dysfunction in MDD.

#### Temporal-occipital network

The reported abnormal connectivities within the temporal-occipital network were in keeping with the inferior longitudinal fasciculus involved in visual emotion and visual memory [48]. The occipital lobe is the visual processing center of the mammalian brain, and the superior occipital cortex is related to object selection [49]. The inferior temporal gyrus is involved in the processing of complex emotional visual stimuli [50] and visual memory [51]. Abnormal connections between the occipital and inferior temporal gyrus would disrupt the occipito-temporal visual system that modulates visual and emotional expertise [52]. Task-related fMRI studies report that depressed patients show abnormal filtering of irrelevant information in the visual cortex [53] and negative biases in visual emotion expression recognition [54], [55]. It was reasonable to speculate that the altered anatomical connectivity in the temporal-occipital network may provide new insights into the abnormal filtering of irrelevant information in MDD.

### Statistical analysis and reliable identification of major depression

**Figure 3:**
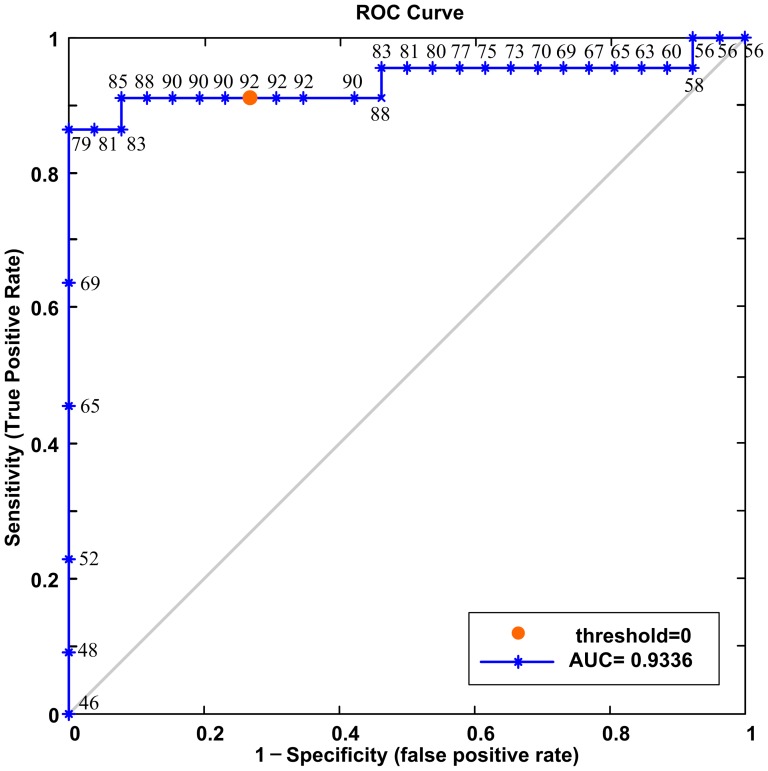
ROC curve of SVM classifier. Numbers around the curve are the correct classification rates (%) corresponding to different sensitivities and specificities. A circular orange point on the curve corresponds to the classification rate, with zero as the classification threshold.

The mean strength for nonzero connectivities of the depressed patients was significantly higher than that of controls. This result indicated that the overall network connectivities of depressed patients were strengthened, which was in partial agreement with previous findings suggesting that depressed patients showed increased function in some regions and networks such as the visual cortical areas [17], default-mode network (DMN) [8], [17], cognitive control network and affective network [19]. Moreover, these significantly altered connections were widely distributed throughout the whole brain, implying that depression was a multi-dimensional and system-level mental disorder [13]. In the present study, a combination of the TSTT, LLE and SVM machine learning approaches was designed to identify altered anatomical connectivity in depressed patients compared with controls. The TSTT and SVM are effective, simple and have been widely used in neuroimaging studies [8], [27], [29], [56], [57], [58]. LLE not only aims at reducing data dimensionality, but also attempts to discover an intrinsic low-dimensional structure of the data [30]. To better understand the contribution that LLE made to the performance of the classifier, we also performed the classification without LLE. The result achieved an accuracy of 85.4%, showing that the LLE method did improve classification performance. The parameters in the SVM and LLE would clearly influence the performance of estimation. In this paper, the parameters have been chosen to maximize the final classification accuracy. We regarded each subject's classifier score as a threshold; the receiver operating characteristics (ROC) curve was determined to further estimate the performance of our classifier (Figure 3). The area under the classifier's ROC curve (AUC) equaled 0.9336, which indicated that our classifier had satisfactory generalization ability. In addition, permutation tests were employed to assess statistical significance of the classification results. Permutation tests validated the relationship between the data and the labels, with a maximum probability of being wrong at 0.0001. In other words, our approaches reliably identified the MDD patients from controls and captured the group differences in the anatomical connectivity patterns; therefore, the anatomical connectivity changes in these networks could be potentially used as a biomarker for MDD.

### Cortical parcellation and definition of connectivity

There are several other types of automatic cortical parcellation methods, such as DICCCOL (Dense Individualized and Common Connectivity-Based Cortical Landmarks) [59], automatic labeling in the Freesurfer [60], random parcellation [61] and the graph theory [62]. Given the lack of a gold standard for cortical parcellation, we utilized the most widely used AAL cortical parcellation method [25], [63], [64], [65], [66]. It has been demonstrated that this cortical parcellation method holds basic connectivity and network properties [65], which is critical for the analysis of network abnormalities discussed in this paper. Many studies define the fiber number between two ROIs as connectivity or use a threshold to construct binary connectivity [65], [66], [67]. Region size differs tremendously in the AAL regions, which means that the total number of fibers reconstructed from each region varies significantly. Because each connection contributed equally to the classification, we normalized the connectivity with the region size. By doing this, we corrected the variable size of cortical ROIs [60], finding it to be helpful with classification. We used the fiber numbers as features for classification, and the results turned out to be poor (GR = 79.2%, SS = 63.6%, SC = 92.3%) compared with our method (GR = 91.7%, SS = 86.4%, SC = 96.2%). These results justified that our definition of connectivity was quite suitable for this study. Although our anatomical network construction seemed to be appropriate for identifying abnormal connections in depressed patients, we still need to investigate the extent to which this construction could be robust to different approaches of cortical parcellation and definition of structural connections.

### Advantages and limitations

Unlike most of the previous DTI studies on MDD that focus on an analysis of intravoxel anisotripy changes [2], [23], [68], [69], the present study takes advantages of a probabilistic tractography technique and regional anatomical connectivity of the whole brain to investigate network abnormalities between depressed patients and controls. There are many advantages to our approaches in DTI processing. First, in contrast to other diffusion tracking studies, we adopt an automatic parcellation method that does not require time-consuming manual selection for specific ROIs, but rather parcellates the cerebral cortex into 90 regions automatically. Second, diffusion tensor tractography allows for the visualization of fiber bundles and provides a tool for estimating WM microstructural and macrostructural characteristics. As a modified diffusion tensor tractograph, the probabilistic tractography technique has many advantages for tracking specific WM tracts in relation to fiber crossing [70]. Moreover, contrary to other voxel-based methods, the probabilistic tractography technique locates the end point of a fiber, which may be crucial when the impact of the disease depends on the origin of these connections [25].

Our current study does have several limitations. First, DTI-based tractography is a relatively new and evolving technique, and it cannot achieve the level of resolution that has been previously obtained by using classic anatomical methods. Without the possibility of distinguishing afferent from efferent pathways using DTI-based tractography, we cannot infer the directionality of reconstructed connections [71]. In addition, using the diffusion images with only fifteen gradient directions limits the angular resolution and therefore increases the uncertainty in fiber tracking [70]. Thus, one must use thirty-two or more directional data in the future. Another limitation of the present study is the small sample size of the test datasets. A small sample size directly impacts the significance level of the TSTT and the generalization rate of the classifier. Therefore, our findings should be confirmed by using a larger sample size in the future.

### Conclusion

In conclusion, this study adds an anatomical connectivity perspective to MDD research and demonstrates that the machine learning approaches can, based on whole-brain anatomical connectivity, identify major depressive individuals from healthy controls with a classification accuracy rate of 91.7%. The most discriminating consensus features show increased anatomical connectivity in the cortical-limbic network of depressed patients. Our results suggest that the altered anatomical connectivity in the cortical-limbic network may contribute to the anatomical basis of emotional dysregulation and cognitive impairments in MDD and may be used as potential biomarkers for MDD diagnoses.

## Materials and Methods

### Ethics Statement

This study was approved by the Ethics Committee of the First Affiliated Hospital of China Medical University. All clinical investigations were conducted according to the principles set forth in the Declaration of Helsinki, and all participants provided written informed consent. Each participant was first informed about the details of the project and then was asked to sign the informed consent form. We confirmed that all potential participants who declined to participate or otherwise did not participate were eligible for treatment (if applicable) and were not disadvantaged in any other way by not participating in this study.

### Participants

The participants consisted of 23 MDD patients from the outpatient clinic at the First Affiliated Hospital of China Medical University who were experiencing a first major depressive episode, and 26 demographically similar healthy controls who were recruited through advertisements. All subjects were right-handed, native Chinese speakers. One depressed patient was removed because we failed to obtain a structural MRI image of the patient. Depressed patients met the criteria for a current episode of unipolar recurrent MDD based on DSM-IV criteria [72]. Clinical psychiatrists diagnosed the subjects as depressed patients through direct interviews using the Structured Clinical Interview for DSM-IV (SCID) [73]. Regarding illnesses, we also excluded participants with a history of head injuries resulting in loss of consciousness and major psychiatric or neurological illness other than depression. None of the subjects had a history of substance abuse or dependence. On the days of the scans, the patients' depressive symptoms were assessed using the 17-item Hamilton Depression Rating Scale (HDRS) [74] and the Clinical Global Impression Scale-Severity (CGI-S) [75]. The remaining 22 MDD patients and 26 healthy controls were matched for age, gender, weight and education. Details regarding both participant groups are shown in Table 2.

**Table 2:** Characteristics of the participants.

Variable	Patient	Control	*p*-value
Sample size	22	26	
Gender (M/F)	7/15	7/19	0.86^a^
Age (years)	31.18±11.05 (19–52)	34.92±9.93 (19–52)	0.54^b^
Education (years)	11.82±3.22	10.76±2.95	0.66^b^
Weight (kg)	60.5±10.93	62.55±8.59	0.45^b^
Number of previous episodes	1.64±0.79		
Duration of current episode (months)	5.68±6.46		
Hamilton Depression Rating Scale (HDRS)	25.95±5.10	4.24±0.99	
Clinical Global Impression Scale-Severity (CGI-S)	5.92±0.65		

a Pearson Chi-square test;

b Two-sample *t*-test.

### Imaging Protocol

DTI scans were acquired using a 1.5T GE Signa Imaging System (General Electric Medical Systems, Milwaukee, Wisconsin, USA) employing a single-shot echo planar imaging sequence. To reduce head movement, the subjects' heads were fixed using foam pads with a standard birdcage head coil. For each slice, 15 images were collected using high diffusion-weighted imaging along 15 non-colinear and non-coplanar directions. The imaging parameters were listed as follows: repetition time (TR) = 12 s; echo time (TE) = 102.4 ms; voxel dimensions = 0.94×0.94×4 mm, scan matrix = 256×256×35, slice thickness = 4 mm, field of view (FOV) = 240×240×140 mm, b value = 1000 sec/mm^2^.

### Region of Interest Segmentation and Fiber Tracking

We used an automatic parcellation method for ROI segmentation and a standard probabilistic tractography algorithm for fiber tracking. ROI segmentation and fiber tracking were all implemented by FSL (http://www.fmrib.ox.ac.uk/fsl) [76]. In contrast to the traditional deterministic-streamline tracking algorithm, the probabilistic algorithm does not simply track WM fibers from voxel to voxel, but also models local diffusion properties and estimates their directions and probabilities. This algorithm generates posterior distributions on the principal direction of diffusion by Markov Chain Monte Carlo (MCMC) sampling and Bayesian inference [25], [70].

**Figure 4:**
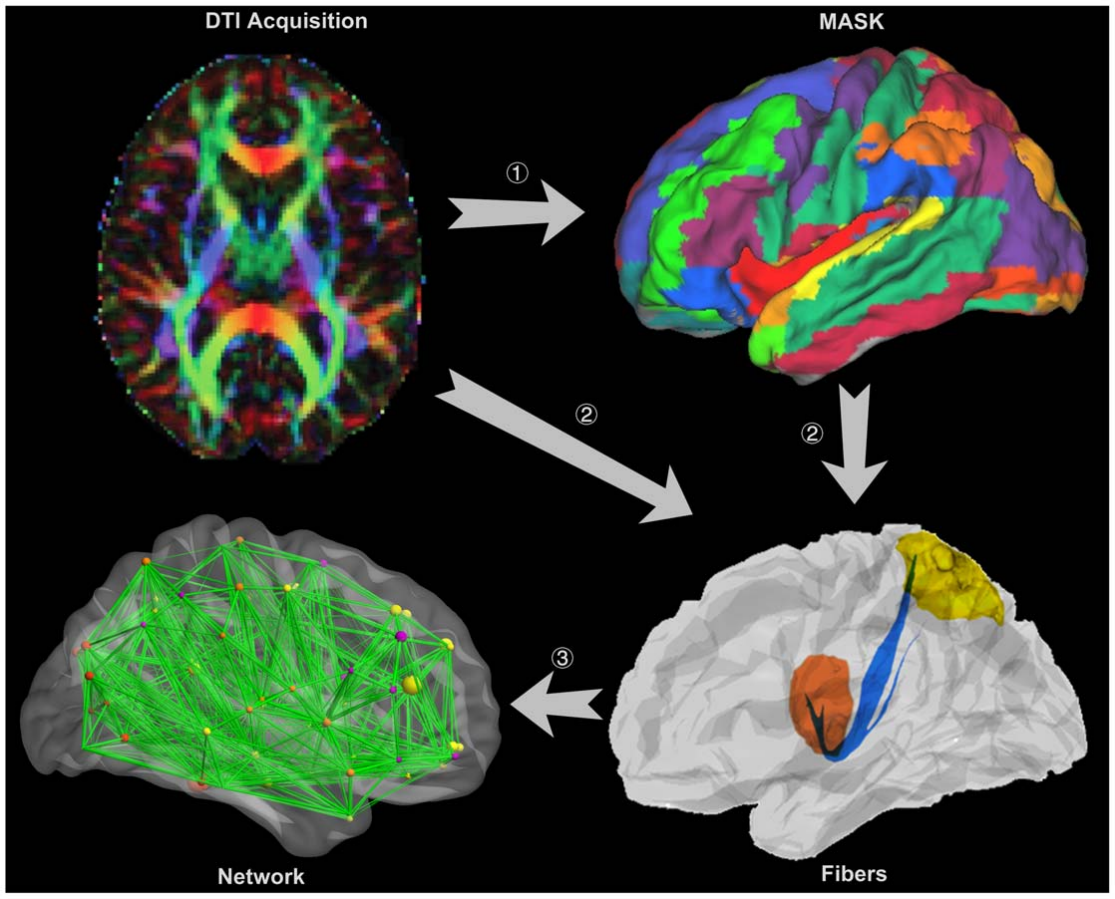
Extraction of a whole brain anatomical network. The DTI image is presented in a reconstructed color-coded tensor map, showing the direction of the principal axis of diffusion using the standard scheme. Blue codes for the superior-inferior, red for left-right, and green for anterior-posterior orientation. (1) Cortical parcellation. The DTI images are mapped with an AAL atlas in the diffusion-MRI native space. (2) Fiber tractography between ROIs. Probabilistic tractography is performed between two ROIs defined in step (1), with only direct connections being retained. (3) Whole brain anatomical network construction. All of the connections in step (2) constitute the whole brain anatomical network.

Extraction of the structural networks was implemented in the following manner, which is displayed graphically in Figure 4:

1. Cortical parcellation. The automated anatomical labeling (AAL) atlas [63] was applied when parcellating the entire cerebral cortex into 90 regions (45 in each hemisphere). First, all images were skullstripped using the FSL Brain Extraction Tool (BET) [77]. Then, the skullstripped T1-weighted MP-RAGE images were registered to the skullstripped b0 image using a 12-parameter affine registration with a mutual information cost function implemented in Flirt (FSL tool) [78] and a nonlinear registration implemented with FNIRT (FSL tool) [79]. Finally, the transformed T1-weighted images were registered to the skullstripped T1 template of ICBM152 in the Montreal Neurological Institute (MNI) space with Flirt, and the resulting transformation matrix was inversed to warp the AAL atlas from the MNI space to the diffusion-MRI native space. In this manner, we obtained an AAL template for each subject (Figure 4, step 1).
2. Interregional connectivity based on probabilistic tractography. The four-dimensional diffusion tensor images were aligned to the first volume using McFlirt (FSL tool) [78] to eliminate head motion error. Then, the aligned diffusion tensor images were corrected for distortions caused by eddy currents by using affine registration in Eddy Current Correction (FSL tool). After completing these preprocesses, a diffusion tensor model was fitted at each voxel using DTIFit (FMRIB Software Library's Diffusion Toolbox) and followed by estimating the local probability distribution of fiber directions at each voxel with BedpostX (FMRIB Software Library's Diffusion Toolbox) [80]. Here, a computation model allowing for automatic estimation of two fiber directions within each voxel was selected to improve the tracking sensitivity of non-dominant fiber populations in the brain [70]. BedpostX generated the basis for probabilistic tractography that was implemented in ProbtrackX (FMRIB Software Library's Diffusion Toolbox). The probabilistic tractography was performed between two ROIs using only direct connections by sampling 5000 streamline fibers with a turning threshold of 60 degrees per voxel (Figure 4, step 2), and then the probabilistic tractography was further constrained to ignore fibers passing through tissue that had a 50% or an even higher probability of being cerebrospinal fluid or gray matter.

By assuming that the *i*-th ROI contained *n* voxels, we seeded 5000 samples at each voxel; therefore, the total number of fibers connected with this ROI was 5000×*n*. Furthermore, if the number of fibers from the *i-*th to the *j-*th ROI was *m*, we obtained the strength of connectivity from the *i-*th to the *j*-th ROI by dividing 5000×*n* by *m* [70]. The fibers estimated from the *i-*th ROI to the *j-*th ROI did not necessarily match the fibers estimated from the *j*-th to *i*-th ROI because seed location affected the probabilistic tractography. The connectivity strength between two regions was defined by averaging these two strengths, and all the connectivity strengths together constituted an anatomical network for the brain (Figure 4, step 3) that was represented in a symmetric 90×90 connectivity matrix (Figure 1 A, B) [81]. Due to low resolution of the DTI images and limitations of the probabilistic tractography, it was inevitable that there were a few false-positive connections between ROIs. Furthermore, the probability of false-positive connections increased when the estimated connectivity strength between the two ROIs was relatively low. A threshold value of 0.01 was applied to reduce false-positive connections between ROIs and to eliminate the connectivities with extraordinarily low strengths [64]. The connectivity derived from this probabilistic tractography has been well-recognized and applied in many neuroimaging studies [64], [81], [82].

### Feature Selection and Classification

First, all the elements in the connection matrix were concatenated to a feature vector and combined as a row in a large feature matrix. Because of noise, low image resolution, registration error and individual differences, the highly discriminating features that account for only a small part of the whole feature matrix are buried by inadequate features. TSTT were applied to identify the significantly different features between groups, which were considered to be the most discriminating. Next, LLE (for more details, see [83]), a manifold learning technique, was introduced to reduce feature space dimensionality to a more manageable level. LLE was chosen because it was capable of obtaining a low-dimensional embedding of the data while preserving the intrinsic data structures [30], [83]. Finally, we adopted SVM with a Gaussian radial basis function kernel for classification.

Classification was performed *N* times with LOOCV. In each fold of LOOCV, one subject was extracted as the test group, and the other *N-1* subjects were retained to train the SVM classifier. First, the most discriminating features were selected from the *N-1* training subjects using TSTT and further projected into the feature space, in which variables between patients and controls were best represented. Then, training samples were used to train the classifier, and test samples were employed to evaluate the classifier performance by comparing classification results with the ground truth class labels. As there are *N* samples, LOOCV trained the classifier *N* times. The performance of a classifier was quantified using Sensitivity (*SS*), Specificity (*SC*) and Generalization Rate (*GR*) based on the results of LOOCV, such that:
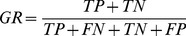
(1)

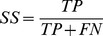
(2)

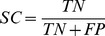
(3)In this case, *TP*, *TN*, *FP* and *FN* represented the number of patients predicted accurately, controls predicted accurately, controls classified as patients and patients classified as controls, respectively. The *SS* indicated the proportion of patients classified correctly, and the *SC* represented the proportion of controls that were classified correctly. The overall proportion of samples classified correctly was represented by *GR*.

To assess the statistical significance of the observed classification accuracy, permutation tests were applied using the generalization rate as the statistic that measured dissimilarity between two populations [84]. The class labels of the training data were first randomly permuted, and then the cross-validation was carried out for each set of label-permuted data. The entire permutation process was repeated 10,000 times [29]. Given the null hypothesis that the classifier could not learn the relationship between the data and the labels reliably when the generalization rate obtained by the classifier trained on the real class labels was lower than the 95% confidence interval of the classifier trained on randomly re-labeled class labels. For any value of the estimated generalization rate *GR*_0_, the appropriate *p*-value *P*(*GR*_0_) represented the probability of observing a classification prediction rate that was no less than *GR*_0_. We were supposed to reject the null hypothesis and declare that the classifier learned the relationship between the data and the labels with a probability of being wrong at most *P*(*GR*_0_).

## References

[1] Drevets WC, Price JL, Furey ML (2008) Brain structural and functional abnormalities in mood disorders: implications for neurocircuitry models of depression. Brain Struct Funct 213: 93–118. 18704495 10.1007/s00429-008-0189-x PMC2522333

[2] Bae JN, Macfall JR, Krishnan KR, Payne ME, Steffens DC, et al (2006) Dorsolateral prefrontal cortex and anterior cingulate cortex white matter alterations in late-life depression. Biol Psychiatry 60: 1356–1363. 16876144 10.1016/j.biopsych.2006.03.052

[3] Drevets WC, Price JL, Bardgett ME, Reich T, Todd RD, et al (2002) Glucose metabolism in the amygdala in depression: relationship to diagnostic subtype and plasma cortisol levels. Pharmacol Biochem Behav 71: 431–447. 11830178 10.1016/s0091-3057(01)00687-6

[4] Videbech P, Ravnkilde B, Pedersen AR, Egander A, Landbo B, et al (2001) The Danish PET/depression project: PET findings in patients with major depression. Psychol Med 31: 1147–1158. 11681541 10.1017/s0033291701004469

[5] Chen CH, Ridler K, Suckling J, Williams S, Fu CHY, et al (2007) Brain imaging correlates of depressive symptom severity and predictors of symptom improvement after antidepressant treatment. Biol Psychiatry 62: 407–414. 17217921 10.1016/j.biopsych.2006.09.018

[6] Drevets WC, Bogers W, Raichle ME (2002) Functional anatomical correlates of antidepressant drug treatment assessed using PET measures of regional glucose metabolism. Eur Neuropsychopharmacol 12: 527–544. 12468016 10.1016/s0924-977x(02)00102-5

[7] Kennedy SH, Evans KR, Kruger S, Mayberg HS, Meyer JH, et al (2001) Changes in regional brain glucose metabolism measured with positron emission tomography after paroxetine treatment of major depression. Am J Psychiatry 158: 899–905. 11384897 10.1176/appi.ajp.158.6.899

[8] Greicius MD, Flores BH, Menon V, Glover GH, Solvason HB, et al (2007) Resting-state functional connectivity in major depression: abnormally increased contributions from subgenual cingulate cortex and thalamus. Biol Psychiatry 62: 429–437. 17210143 10.1016/j.biopsych.2006.09.020 PMC2001244

[9] Tae WS, Kim SS, Lee KU, Nam EC, Kim KW (2008) Validation of hippocampal volumes measured using a manual method and two automated methods (FreeSurfer and IBASPM) in chronic major depressive disorder. Neuroradiology 50: 569–581. 18414838 10.1007/s00234-008-0383-9

[10] Ballmaier M, Toga AW, Blanton RE, Sowell ER, Lavretsky H, et al (2004) Anterior cingulate, gyrus rectus, and orbitofrontal abnormalities in elderly depressed patients: an MRI-based parcellation of the prefrontal cortex. Am J Psychiatry 161: 99–108. 14702257 10.1176/appi.ajp.161.1.99

[11] Tang YQ, Wang F, Xie GR, Liu J, Li LH, et al (2007) Reduced ventral anterior cingulate and amygdala volumes in medication-naive females with major depressive disorder: a voxel-based morphometric magnetic resonance imaging study. Psychiatry Res-Neuroim 156: 83–86. 10.1016/j.pscychresns.2007.03.005 17825533

[12] Hickie IB, Naismith SL, Ward PB, Scott EM, Mitchell PB, et al (2007) Serotonin transporter gene status predicts caudate nucleus but not amygdala or hippocampal volumes in older persons with major depression. J Affect Disord 98: 137–142. 16930719 10.1016/j.jad.2006.07.010

[13] Mayberg HS (1997) Limbic-cortical dysregulation: a proposed model of depression. J Neuropsychiatry Clin Neurosci 9: 471–481. 9276848 10.1176/jnp.9.3.471

[14] Mayberg HS (2003) Modulating dysfunctional limbic-cortical circuits in depression: towards development of brain-based algorithms for diagnosis and optimised treatment. Br Med Bull 65: 193–207. 12697626 10.1093/bmb/65.1.193

[15] Linden DE, Fallgatter AJ (2009) Neuroimaging in psychiatry: from bench to bedside. Front Hum Neurosci 3: 49. 20087437 10.3389/neuro.09.049.2009 PMC2807751

[16] Bennett MR (2011) The prefrontal-limbic network in depression: modulation by hypothalamus, basal ganglia and midbrain. Prog Neurobiol 93 (4) 468–487. 21349315 10.1016/j.pneurobio.2011.01.006

[17] Zeng L-L, Shen H, Liu L, Wang L, Li B, et al (2012) Identifying major depression using whole-brain functional connectivity: a multivariate pattern analysis. Brain 135: 1498–1507. 22418737 10.1093/brain/aws059

[18] Liu L, Zeng L-L, Li Y, Ma Q, Li B, et al (2012) Altered cerebellar functional connectivity with intrinsic connectivity networks in adults with major depressive disorder. PLoS ONE 7 (6) e39516. 22724025 10.1371/journal.pone.0039516 PMC3377654

[19] Sheline YI, Price JL, Yan ZZ, Mintun MA (2010) Resting-state functional MRI in depression unmasks increased connectivity between networks via the dorsal nexus. Proc Natl Acad Sci USA 107: 11020–11025. 20534464 10.1073/pnas.1000446107 PMC2890754

[20] Amelia V, Jorge RC, Hassel S (2008) Elevated left and reduced right orbitomedial prefrontal fractional anisotropy in adults with bipolar disorder revealed by tract-based spatial statistics. Arch Gen Psychiatry 65 (9) 1041–1052. 18762590 10.1001/archpsyc.65.9.1041 PMC2730162

[21] Zhang D, Guo L, Hu X, Li K, Zhao Q, et al (2012) Increased cortico-subcortical functional connectivity in schizophrenia. Brain Imaging Behav 6: 27–35. 22002475 10.1007/s11682-011-9138-z

[22] Taylor WD, Kuchibhatla M, Payne ME, Macfall JR, Sheline YI, et al (2008) Frontal white matter anisotropy and antidepressant remission in late-life depression. PLoS ONE 3: e3267. 18813343 10.1371/journal.pone.0003267 PMC2533397

[23] Nobuhara K, Okugawa G, Minami T, Takase K, Yoshida T, et al (2004) Effects of electroconvulsive therapy on frontal white matter in late-life depression: a diffusion tensor imaging study. Neuropsychobiology 50: 48–53. 15179020 10.1159/000077941

[24] Houenou J, Wessa M, Douaud G, Leboyer M, Chanraud S, et al (2007) Increased white matter connectivity in euthymic bipolar patients: diffusion tensor tractography between the subgenual cingulate and the amygdalo-hippocampal complex. Mol Psychiatry 12: 1001–1010. 17471288 10.1038/sj.mp.4002010

[25] Robinson EC, Hammers A, Ericsson A, Edwards D, Rueckert D (2010) Identifying population differences in whole-brain structural networks: a machine learning approach. NeuroImage 50: 910–919. 20079440 10.1016/j.neuroimage.2010.01.019

[26] Fan Y, Wu X, Davatzikos C (2008) Structural and functional biomarkers of prodromal Alzheimer's disease: a high-dimensional pattern classification study. NeuroImage 41: 277–285. 18400519 10.1016/j.neuroimage.2008.02.043 PMC2682533

[27] Costafreda SG, Chu C, Ashburner J, Fu CHY (2009) Prognostic and diagnostic potential of the structural neuroanatomy of depression. PLoS ONE 4: e6353. 19633718 10.1371/journal.pone.0006353 PMC2712086

[28] Fu CHY, Mourao-Miranda J, Costafrecla SG, Khanna A, Marquand AF, et al (2008) Pattern classification of sad facial processing: toward the development of neurobiological markers in depression. Biol Psychiatry 63: 656–662. 17949689 10.1016/j.biopsych.2007.08.020

[29] Dosenbach NU, Nardos B, Cohen AL, Fair DA, Power JD, et al (2010) Prediction of individual brain maturity using fMRI. Science 329: 1358–1361. 20829489 10.1126/science.1194144 PMC3135376

[30] Shen H, Wang LB, Liu YD, Hu DW (2010) Discriminative analysis of resting-state functional connectivity patterns of schizophrenia using low dimensional embedding of fMRI. NeuroImage 49: 3110–3121. 19931396 10.1016/j.neuroimage.2009.11.011

[31] Sheline YI (2003) Neuroimaging studies of mood disorder effects on the brain. Biol Psychiatry 54: 338–352. 12893109 10.1016/s0006-3223(03)00347-0

[32] Ochsner KN, Gross JJ (2005) The cognitive control of emotion. Trends Cogn Sci 9: 242–249. 15866151 10.1016/j.tics.2005.03.010

[33] Drevets WC (2001) Neuroimaging and neuropathological studies of depression: implications for the cognitive-emotional features of mood disorders. Curr Opin Neurobiol 11: 240–259. 11301246 10.1016/s0959-4388(00)00203-8

[34] Frodl T, Bokde ALW, Scheuerecker J, Lisiecka D, Schoepf V, et al (2010) Functional connectivity bias of the orbitofrontal cortex in drug-free patients with major depression. Biol Psychiatry 67: 161–167. 19811772 10.1016/j.biopsych.2009.08.022

[35] Phillips ML, Drevets WC, Rauch SL, Lane R (2003) Neurobiology of emotion perception II: implications for major psychiatric disorders. Biol Psychiatry 54: 515–528. 12946880 10.1016/s0006-3223(03)00171-9

[36] Alexopoulos GS, Murphy CF, Gunning-Dixon FM, Latoussakis V, Kanellopoulos D, et al (2008) Microstructural white matter abnormalities and remission of geriatric depression. Am J Psychiatry 165: 238–244. 18172016 10.1176/appi.ajp.2007.07050744

[37] Grace AA (2006) Disruption of cortical-limbic interaction as a substrate for comorbidity. Neurotoxicity Research 10 (2) 93–101. 17062371 10.1007/BF03033238

[38] Samantha JB, Charmaine D, Stefan D, Suzannah KH, Christopher JJ, et al (2009) Default-mode brain dysfunction in mental disorders: a systematic review. Neurosci Biobehav Rev 279–296. 18824195 10.1016/j.neubiorev.2008.09.002

[39] Rizzolatti G, Fogassi L, Gallese V (1997) Parietal cortex: from sight to action. Current Opinion in Neurobiology 7: 562–567. 9287198 10.1016/s0959-4388(97)80037-2

[40] Behrmann M, Geng JJ, Shomstein S (2004) Parietal cortex and attention. Current Opinion in Neurobiology 14: 212–217. 15082327 10.1016/j.conb.2004.03.012

[41] Paulus MP, Hozack NE, Zauscher BE, Frank L, Brown GG, et al (2002) Parietal Dysfunction Is Associated with Increased Outcome-Related Decision-Making in Schizophrenia Patients. Biol Psychiatry 51: 995–1004. 12062884 10.1016/s0006-3223(01)01358-0

[42] Mayberg HS, Liotti M, Brannan SK, McGinnis S, Mahurin RK, et al (1999) Reciprocal limbic-cortical function and negative mood: converging PET findings in depression and normal sadness. Am J Psychiatry 156 (5) 675–682. 10327898 10.1176/ajp.156.5.675

[43] Seminowicz DA, Mayberg HS, McIntosh AR, Goldapple K, Kennedy S, et al (2004) Limbic-frontal circuitry in major depression: a path modeling metanalysis. NeuroImage 22: 409–418. 15110034 10.1016/j.neuroimage.2004.01.015

[44] Price JL, Drevets WC (2010) Neurocircuitry of mood disorders. Neuropsychopharmacology 35: 192–216. 19693001 10.1038/npp.2009.104 PMC3055427

[45] Ebmeier KP, Donaghey C, Steele JD (2006) Recent developments and current controversies in depression. Lancet 367: 153–167. 16413879 10.1016/S0140-6736(06)67964-6

[46] Salmond CH, Menon DK, Chatfield DA, Williams GB, Pena A, et al (2006) Diffusion tensor imaging in chronic head injury survivors: Correlations with learning and memory indices. NeuroImage 29: 117–124. 16084738 10.1016/j.neuroimage.2005.07.012

[47] Honey CJ, Sporns O, Cammoun L, Gigandet X, Thiran JP, et al (2009) Predicting human resting-state functional connectivity from structural connectivity. Proc Natl Acad Sci USA 106: 2035–2040. 19188601 10.1073/pnas.0811168106 PMC2634800

[48] Catani M, Jones DK, Donato R, ffytche DH (2003) Occipito-temporal connections in the human brain. Brain 126: 2093–2107. 12821517 10.1093/brain/awg203

[49] Grill-Spector K, Malach R (2004) The human visual cortex. Annu Rev Neurosci 27: 649–677. 15217346 10.1146/annurev.neuro.27.070203.144220

[50] Geday J, Ehlersf L, Boldsen AS, Gjedde A (2001) The inferior temporal and orbitofrontal cortex in analysing emotional pictures. NeuroImage 13: 6.

[51] Masahiko M, Atsuo S (2002) Computational modeling of pair-association memory in inferior temporal cortex. Cogn Brain Res 13: 169–178. 10.1016/s0926-6410(01)00109-4 11958959

[52] Krolak-Salmon P, Hénaff M-A, Vighetto A, Bertrand O, Mauguière F (2004) Early amygdala reaction to fear spreading in occipital, temporal, and frontal cortex: A depth electrode ERP study in human. Neuron 42: 665–676. 15157426 10.1016/s0896-6273(04)00264-8

[53] Desseilles M, Balteau E, Sterpenich V, Dang-Vu TT, Darsaud A, et al (2009) Abnormal neural filtering of irrelevant visual information in depression. J Neurosci 29: 1395–1403. 19193886 10.1523/JNEUROSCI.3341-08.2009 PMC6666064

[54] Surguladze S, Brammer MJ, Keedwell P, Giampietro V, Young AW, et al (2005) A differential pattern of neural response toward sad versus happy facial expressions in major depressive disorder. Biol Psychiatry 57 (3) 201–209. 15691520 10.1016/j.biopsych.2004.10.028

[55] Keedwell PA, Andrew C, Williams SCR, Brammer MJ, Phillips ML (2005) A double dissociation of ventromedial prefrontal cortical responses to sad and happy stimuli in depressed and healthy individuals. Biol Psychiatry 58: 495–503. 15993859 10.1016/j.biopsych.2005.04.035

[56] Wang L, Shen H, Tang F, Zang Y, Hu D (2012) Combined structural and resting-state functional MRI analysis of sexual dimorphism in the young adult human brain: An MVPA approach. NeuroImage 61: 931–940. 22498657 10.1016/j.neuroimage.2012.03.080

[57] Liu M, Zeng L-L, Shen H, Liu Z, Hu D (2012) Potential risk for healthy siblings to develop schizophrenia: evidence from pattern classification with whole-brain connectivity. NeuroReport 23: 265–269. 22158134 10.1097/WNR.0b013e32834f60a5

[58] Grimm S, Boesiger P, Beck J, Schuepbach D, Bermpohl F, et al (2009) Altered negative BOLD responses in the default-mode network during emotion processing in depressed subjects. Neuropsychopharmacology 34: 932–943. 18536699 10.1038/npp.2008.81

[59] Zhu D, Li K, Guo L, Jiang X, Zhang T, et al (2012) DICCCOL: Dense Individualized and Common Connectivity-Based Cortical Landmarks. Cerebral Cortex In press. 10.1093/cercor/bhs072 PMC3593574 22490548

[60] Hagmann P, Cammoun L, Gigandet X, Meuli R, Honey CJ, et al (2008) Mapping the structural core of human cerebral cortex. PLoS Biol 6 (7) e159. 18597554 10.1371/journal.pbio.0060159 PMC2443193

[61] Hagmann P, Kurant M, Gigandet X, Thiran P, Wedeen VJ, et al (2007) Mapping human whole-brain structural networks with diffusion MRI. PLoS ONE 2 (7) e597. 17611629 10.1371/journal.pone.0000597 PMC1895920

[62] Iturria-Medina Y, Canales-Rodríguez EJ, Melie-García L, Valdés-Hernández PA, Martínez-Montes E, et al (2007) Characterizing brain anatomical connections using diffusion weighted MRI and graph theory. NeuroImage 36: 645–660. 17466539 10.1016/j.neuroimage.2007.02.012

[63] Tzourio-Mazoyer N, Landeau B, Papathanassiou D, Crivello F, Etard O, et al (2002) Automated anatomical labeling of activations in SPM using a macroscopic anatomical parcellation of the MNI MRI single-subject brain. NeuroImage 15: 273–289. 11771995 10.1006/nimg.2001.0978

[64] Gong G, Rosa-Neto P, Carbonell F, Chen ZJ, He Y, et al (2009) Age- and gender-related differences in the cortical anatomical network. J Neurosci 29 (50) 15684–15693. 20016083 10.1523/JNEUROSCI.2308-09.2009 PMC2831804

[65] Bassett DS, Brown JA, Deshpande V, Carlson JM, Grafton ST (2011) Conserved and variable architecture of human white matter connectivity. NeuroImage 54: 1262–1279. 20850551 10.1016/j.neuroimage.2010.09.006

[66] Zalesky A, Fornito A, Harding IH, Cocchi L, Yücel M, et al (2010) Whole-brain anatomical networks: does the choice of nodes matter? NeuroImage 50: 970–983. 20035887 10.1016/j.neuroimage.2009.12.027

[67] Liu T (2011) A few thoughts on brain ROIs. Brain Imaging Behav 5: 189–202. 21556745 10.1007/s11682-011-9123-6 PMC3927780

[68] McLaughling NCR, Paul RH, Grieve SM, Williams LM, Laidlaw D, et al (2007) Diffusion tensor imaging of the corpus callosum: a cross-sectional study across the lifespan. Int J Devl Neuroscience 25: 215–221. 10.1016/j.ijdevneu.2007.03.008 17524591

[69] Versace A, Almeida JRC, Quevedo K, Thompson WK, Terwilliger RA, et al (2010) Right orbitofrontal corticolimbic and left corticocortical white matter connectivity differentiate bipolar and unipolar depression. Biol Psychiatry 68: 560–567. 20598288 10.1016/j.biopsych.2010.04.036 PMC3743239

[70] Behrens TE, Johansen BH, Jbabdi S, Rushworth MF, Woolrich MW (2007) Probabilistic diffusion tractography with multiple fibre orientations: what can we gain?. NeuroImage 34: 144–155. 17070705 10.1016/j.neuroimage.2006.09.018 PMC7116582

[71] Gutman DA, Holtzheimer PE, Behrens TE, Johansen BH, Mayberg HS (2009) A tractography analysis of two deep brain stimulation white matter targets for depression. Biol Psychiatry 65: 276–282. 19013554 10.1016/j.biopsych.2008.09.021 PMC4423548

[72] APA (2000) Diagnostic and statistical manual of mental disorders(4th edition). Washington, DC: American Psychiatric Press.

[73] First MB, Spitzer RL, Gibbon M (1995) Structured clinical interview for DSM-IV axis 1 disorder-patient edition(SCID-I/P). New York: New York State Psychiatric Institute.

[74] Hamilton M (1960) A rating scale for depression. J Neurol Neurosurg Psychiatry 23: 56–62. 14399272 10.1136/jnnp.23.1.56 PMC495331

[75] Guy W (1976) Clinical global impressions: in ECDEU assessment manual for psychopharmacology. Revised DHEW Pub. (ADM). Rockville,MD: National Institute for Mental Health. 218–222 p.

[76] Smith SM, Jenkinson M, Woolrich MW, Beckmann CF, Behrens TE, et al (2004) Advances in functional and structural MR image analysis and implementation as FSL. NeuroImage 23: 208–219. 10.1016/j.neuroimage.2004.07.051 15501092

[77] Smith SM (2002) Fast robust automated brain extraction. Human Brain Mapping 17 (3) 143–155. 12391568 10.1002/hbm.10062 PMC6871816

[78] Jenkinson M, Bannister P, Brady M, Smith SM (2002) Improved optimization for the robust and accurate linear registration and motion correction of brain images. NeuroImage 17 (2) 825–841. 12377157 10.1016/s1053-8119(02)91132-8

[79] Andersson J, Jenkinson M, Smith SM (2007) Non-linear registration, aka Spatial normalisation. FMRIB technical report TR07JA2.

[80] Behrens TE, Woolrich MW, Jenkinson M, Johansen BH, Nunes RG, et al (2003) Characterization and propagation of uncertainty in diffusion-weighted MR imaging. Magn Reson Med 50: 1077–1088. 14587019 10.1002/mrm.10609

[81] Behrens TE, Johansen BH, Woolrich MW, Smith SM, Wheeler-Kingshott CA, et al (2003) Non-invasive mapping of connections between human thalamus and cortex using diffusion imaging. Nat Neuroscience 6: 750–757. 12808459 10.1038/nn1075

[82] Johansen-Berg H, Behrens TE, Robson MD, Drobnjak I, Rushworth MF, et al (2004) Changes in connectivity profiles define functionally distinct regions in human medial frontal cortex. Proc Natl Acad Sci USA 101: 13335–13340. 15340158 10.1073/pnas.0403743101 PMC516567

[83] Roweis ST, Saul LK (2000) Nonlinear dimensionality reduction by locally linear embedding. Science 290: 2323–2326. 11125150 10.1126/science.290.5500.2323

[84] Golland P, Fischl B (2003) Permutation tests for classification: towards statistical significance in image-based studies. Inf Process Med Imaging 2732: 330–341. 10.1007/978-3-540-45087-0_28 15344469

